# Non-motor symptoms fluctuations in patients with Parkinson's disease at the Clinical Hospital of Salvador, Bahia

**DOI:** 10.1590/1980-5764-DN-2021-0056

**Published:** 2022-04-29

**Authors:** Karollyne Santos Barreto, Jamary Oliveira, Luana Dias Reis, Tayane Guimarães Ribeiro, Roberta Borges Gomes Kauark

**Affiliations:** 1 Universidade Federal da Bahia, Faculdade de Medicina da Bahia, Salvador BA, Brazil.; 2 Complexo Hospitalar Universitário Professor Edgard Santos, Salvador BA, Brazil.; 3 Escola Bahiana de Medicina, Salvador BA, Brazil.

**Keywords:** Parkinson Disease, Symptom Assessment, Doença de Parkinson, Avaliação de Sintomas

## Abstract

Motor fluctuations in Parkinson's disease (PD) are a frequent long-term complication. Knowledge is limited on the prevalence and incidence of non-motor symptoms (NMS) fluctuations, especially in Brazil.

**Objective:**

The objective of this study was to verify the frequency of NMS fluctuations and its relationship with other aspects of PD in patients followed at an outpatient movement disorders clinic.

**Methods:**

This is a cross-sectional study in which patients were evaluated for the presence of both types of fluctuations using the Wearing Off Questionnaire (WOQ-19).

**Results:**

A total of 37 patients (11 women and 26 men) were participated in this study, and the frequency of NMS fluctuations was 54.1% (90.9% in women and 38.5% in men). Anxiety was the most frequent non-motor fluctuation (35.1%). The highest percentage of NMS fluctuations (70%) was found in the group in which disease duration was more than 6 years. Most patients with motor fluctuations also had NMS fluctuations (66.7%). No patient presented with isolated NMS fluctuations.

**Conclusions:**

This study showed that, in the study population, approximately half of the patients had NMS fluctuations, with a higher frequency among women. A higher frequency was present in patients with earlier age of diagnosis, longer duration, and greater severity of disease. These findings point to the importance of recognizing the fluctuations of NMS in the study population, since these may not be spontaneously mentioned by the patient, who is remaining unnoticed, undiagnosed, and not treated by the neurologist, representing a significant aggravating factor in the patient's quality of life.

## RESUMO.

A flutuação dos sintomas na doença de Parkinson é uma complicação frequente em longo prazo. Pouco é conhecido sobre a prevalência e a incidência de flutuações de sintomas não motores (SNM), principalmente na população brasileira.

**Objetivo:**

Verificar a frequência das flutuações dos SNM e sua relação com outros aspectos da doença de Parkinson em pacientes acompanhados em ambulatório de movimentos anormais do Hospital das Clínicas de Salvador, Bahia.

**Métodos:**

Trata-se de um estudo de corte transversal, no qual os pacientes foram avaliados quanto à presença de flutuações utilizando-se o Wearing-Off Questionnaire (WOQ-19).

**Resultados:**

O total de 37 pacientes (11 mulheres e 26 homens) participou do estudo. A frequência de flutuações dos SNM foi de 54,1% (90,9% no sexo feminino e 38,5% no sexo masculino). Ansiedade foi a flutuação não motora mais frequente (35,1%). O maior percentual de flutuações dos SNM (70%) encontrou-se no grupo cujo tempo de doença estava acima de seis anos. A maioria dos pacientes com flutuações motoras teve também flutuações dos SNM (66,7%). Nenhum paciente apresentou apenas flutuação de SNM.

**Conclusões:**

O presente estudo mostrou que, na população estudada, aproximadamente metade dos pacientes apresentaram flutuações dos SNM, sendo essa frequência maior no sexo feminino. Estes achados apontam para a importância do reconhecimento das flutuações dos SNM na população estudada, já que elas podem não ser espontaneamente citadas pelo paciente, passando despercebidas, não sendo diagnosticadas nem tratadas pelo neurologista e constituindo-se em agravante não desprezível na qualidade de vida dos pacientes.

## INTRODUCTION

Parkinson's disease (PD) is the second most common neurodegenerative disorder worldwide^1^. Although the disease is widely known for its motor symptoms (MS), the involvement of structures outside the basal nuclei circuit is quite significant, and the resulting changes form a set of symptoms called non-MS (NMS). Psychiatric-behavioral disorders, autonomic dysfunctions, and sensory symptoms are among the most common non-motor manifestations^2^. These changes may precede the onset of MS and continue to progress after the diagnosis of PD, following the patients throughout their lifetime^3^.

Treatment with dopamine replacement is very effective during the first year of the disease, significantly decreasing the appearance of MS^4^. However, with the progress of dopaminergic neuronal degeneration and long-term treatment, complications begin to emerge, one of them being the shortening duration of the medication effect. As a result, parkinsonian symptoms, once suppressed by therapy, start to manifest in different levels before the next dose is taken. This phenomenon is also known as end-of-dose deterioration or wearing-off^2^. The appearance of fluctuations in symptoms is so common that, in their absence, a reassessment of the diagnosis of PD should be performed.

Motor fluctuations are extensively studied. However, concerning non-motor fluctuations, although already described, there are still many gaps in the literature regarding their incidence and prevalence in the population with PD, as well as the impact on the quality of life of these patients. Nevertheless, its presence significantly impairs patients’ quality of life and can be as harmful as or even worse than motor fluctuations^4^. Consistent studies have shown that NMS, such as depression, anxiety, sleep disorders, and fatigue, were primarily responsible for the worst quality of life scores. In these studies, the motor component played a minor role in worsening the patient's quality of life, showing the importance of giving attention to NMS and its fluctuations^5,6^.

There are very few population-based studies that describe the prevalence of PD in the Brazilian population. In one of these studies, a prevalence of 3.3% was found in individuals aged above 64 years^7^. This study did not address the aspects related to fluctuations of NMS. Therefore, studies carried out on Brazilian PD population are strongly relevant, especially on such a poorly explored theme as non-motor fluctuations.

The goal of this study was to verify the frequency of non-motor fluctuations and their relationship with other epidemiological and clinical aspects of PD in a sample of patients from the outpatient clinic of movement disorders of the Edgar Santos Professor University Hospital (HUPES). Data obtained have the potential to contribute to a better understanding of the characteristics of non-motor fluctuations in patients with PD followed at the mentioned outpatient clinic.

## METHODS

This study is a cross-sectional study, in which data from individuals who were diagnosed with PD and followed up at an outpatient clinic in the city of Salvador (Bahia) were collected during the period from June to October 2018. It was a convenience sample, which was composed of all the interviews performed during this period.

To be included in the research, individuals had to have a diagnosis of PD given by a neurologist who specialized in movement disorders (according to the Queen Square Brain Bank criteria)^8^, the absence of dementia of any other etiologies (except dementia in PD) supported by laboratory and imaging tests, 18 years of age or older, and to agree to participate in this study. Individuals with parkinsonism associated with other neurodegenerative diseases or secondary parkinsonism, and individuals with impaired hearing or visual deficit were excluded from this study.

Initially, 90 patients who attended the movement disorders outpatient clinic were screened. Of these, 28 patients were excluded because they had a diagnosis of parkinsonism associated with other neurodegenerative diseases or secondary parkinsonism. Of the 62 remaining patients with PD, data were collected from 37 of them, when they attended the routine appointment. The other 25 patients with PD did not participate in this study for several reasons: refusal to participate, not attending the routine appointment, impossibility of interviewing them on routine appointment day due to the limited number of researchers and/or attendance room, severe auditory or visual deficits, or dementia of other etiologies other than dementia in PD.

Demographic data were obtained in the interview through a standardized questionnaire, complemented by the patient's medical record. Clinical and functional data were obtained through the application of the Unified Parkinson's Disease Scale (UPDRS) Part III^9^ and the Hoehn and Yahr Scale (H&Y)^10^. Data related to NMS and NMS fluctuations were obtained from the application of the Non-Motor Symptom Assessment Scale (NMAS) for PD^11^ and the Wearing Off Questionnaire (WOQ-19)^12^, respectively. All the scales were filled by the researcher during the interview to the individual and by clinical examination and observation.

The presence of fluctuations in symptoms was determined by the WOQ-19, which consists of 19 items, 9 of them addressing MS and 10 addressing NMS. For each item, the patients were questioned about the presence of the symptom and its improvement after the next dose of dopaminergic treatment. The positive response to the last question, of any symptom, identified the patient as belonging to the group with general fluctuation of symptoms (WOQ-19 total score of ≥1)^12^. Patients were considered to have MS fluctuations when a cutoff score of ≥1 for MS of the WOQ-19 was reached (WOQ-19 M≥1). Likewise, patients were considered to have NMS fluctuations when a cutoff score of ≥1 for NMS of the WOQ-19 was reached (WOQ-19 NM≥1). The WOQ-19 has not yet been formally validated in Brazil, and, therefore, its items were freely translated by the research team to the Portuguese language.

## Statistical analysis plan

For the elaboration of the database and descriptive analysis, the software Statistical Package for the Social Sciences (SPSS Inc., Chicago, IL, USA), version 17.0, for Windows was used. The results were presented using tables. Categorical variables were expressed as frequencies and percentages — n (%). Continuous variables with normal distribution were expressed as means and standard deviations, and those with non-normal distribution were expressed in median and interquartile range. The normality of the numerical variables was verified through descriptive statistics, graphical analysis, and the Kolmogorov-Smirnov test.

When comparing categorical variables, the χ^2^ test was used. When the distribution had an “n” in each category containing less than five individuals, Fischer's exact test was used. For the comparison between categorical variables (two groups: absence and presence of NMS fluctuations) and numerical variables, the independent sample Student's *t*-test was used when the variables had a normal distribution, and the Mann-Whitney U test was used for those with an asymmetrical distribution.

### Ethical considerations

The survey subjects had no research costs. All patients were signed the informed consent form prior to data collection.

This work composes an arm of a larger research, previously approved by the Research Ethics Committee (CEP) of the HUPES Complex, with an approval issued on May 18, 2018, and CAAE number 82741418.6.0000.0049.

## RESULTS

This study consisted of 37 patients (11 women and 6 men). Demographic and clinical characteristics are shown in Table 1.

**Table 1:** Demographic and clinical characteristics of the sample.

General features		
Age in years mean (SD)		62.08 (7.41)
Age of onset in years mean (SD)		54.46 (8.51)
Duration of disease in years mean (SD)		7.62 (5.10)
Part III UPDRS mean (SD)		44.59 (16.28)
Hoehn & Yahr* mean (SD)		3.0 (3.0–2.0)
Gender n (%)	Male	26 (70.3%)
Color n (%)	White	5 (13.51%)
	Brown	21 (56.8%)
	Black	11 (29.7%)
Scholarity n (%)	Illiterate	5 (13.5%)
	Elementary school (complete or incomplete)	14 (37.8%)
	High school (complete or incomplete)	15 (40.5%)
	College (complete or incomplete or postgraduate)	3 (8.1%)

SD: standard deviation; UPDRS: Unified Parkinson's Disease Assessment Scale;

* median (interquartile range).

According to the cutoff point of the WOQ-19 total score, 30 (81.5%) patients were identified with general fluctuation of symptoms (Table 2). The frequency of NMS fluctuations in the sample was 54.1%. The frequency of NMS fluctuations was 90.9% in women and 38.5% in men. Anxiety was the most frequent NMS fluctuation (35.1%) diagnosed in the whole sample, and also the most frequent non-motor fluctuation (63.3%) in women. In men, anxiety (23.1%) and mood changes (23.1%) were among the most frequent NMS fluctuations. In both, there were no panic attacks diagnosed as a non-motor fluctuation.

**Table 2:** Frequency of non-motor symptoms fluctuations according to the Wearing Off Questionnaire.

Symptoms, n (%)	All (37)	Women (11)	Men (26)	p-value
Anxiety	13 (35.1%)	7 (63.3%)	6 (23.1%)	0.035 §
Experiencing sweating	6 (16.2%)	4 (36.4%)	2 (7.7%)	0.051||
Mood changes	9 (24.3%)	3 (27.3%)	6 (23.1%)	0.672||
Numbness	9 (24.3%)	4 (36.4%)	5 (19.2%)	0.267§
Experiencing panic attacks	0	0	0	—
Cloudy mind/dullness thinking	5 (13.5%)	4 (36.4%)	1 (3.8%)	0.021||
Abdominal discomfort	2 (5.4%)	1 (9.1%)	1 (3.8%)	0.512||
Experiencing hot and cold	4 (10.8%)	3 (27.3%)	1 (3.8%)	0.070||
Pain	8 (21.6%)	4 (36.4%)	4 (15.4%)	0.404||
WOQ-19 total score ≥1*	30 (81.5%)	10 (90.9%)	20 (76.9%)	0.649||
WOQ-19 NM ≥1+	20 (54.1%)	10 (90.9%)	10 (38.5%)	0.004||

* Frequency of general fluctuation of symptoms;

+ frequency of NMS fluctuations;

§ χ^2^ test;

|| Fischer's exact test.

NMS: non-motor symptoms.

Table 3 shows the comparison of the frequency of NMS fluctuation between groups divided by the median age at diagnosis and the duration of the disease. The association between the younger age of the diagnosis and the presence of NMS fluctuation stands out, and this association was possible to notice by assessing the age of the diagnosis in both categorical (below 54 years old) and numerical ways. For the variable disease duration, an association is observed between longer disease duration and presence of NMS fluctuation (both in the numerical and categorical variables).

**Table 3:** Comparison of the frequency of non-motor symptoms fluctuation between groups divided by the median age at diagnosis and disease duration.

	Median	NMS fluctuations n (%)			p-value
		WOQ-19 NM <1*	WOQ-19 NM ≥1+	Total	
Diagnostic age	<54 years	4 (23.5%)	15 (78.9%)	19 (51.4%)	
	≥54 years	13 (76.5%)	5 (27.8%)	18 (48.6%)	0.003§
	Total	17 (100%)	20 (100%)	37 (100%)	
Diagnostic age		58.8±6.9	49.7±8.4		0.001||
Diagnostic age (numeric variable)		63.5±7.2	59.8±7.9		0.140||
Disease duration	≤6 years	14 (82.4%)	6 (30%)	20 (54.1%)	0.003§
	>6 years	3 (17.6%)	14 (70%)	17 (45.9%)	
Disease duration (numeric variable)		4 (2–5.5)	9.5 (6.0–14.5)		0.001¶
Total		17 (100%)	20 (100%)	37 (100%)	

* Absence of NMS fluctuations;

+ presence of NMS fluctuations;

§ Fischer's exact test;

|| independent sample Student's *t*-test;

¶ Mann-Whitney U test.

NMS: non-motor symptoms.

**Figure 1:**
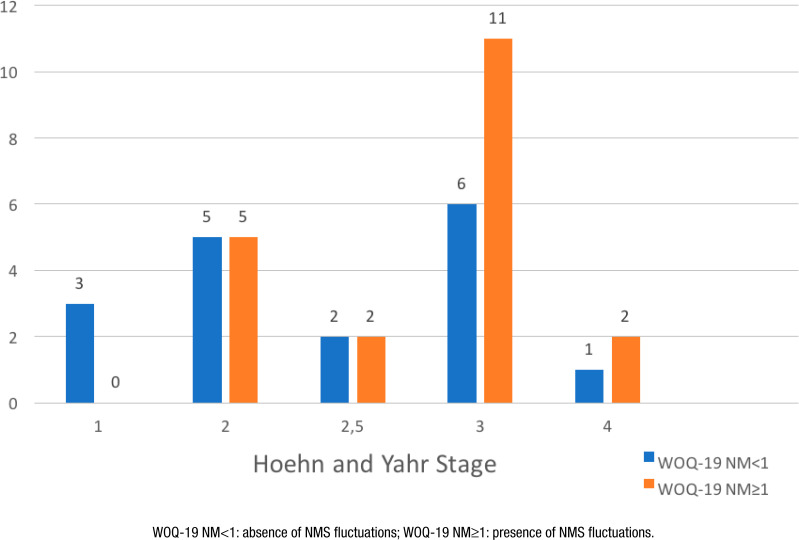
Comparison of the frequency of non-motor symptoms fluctuation and disease severity (H&Y).

The frequency of NMS fluctuation was analyzed according to the severity of the disease, as verified by the H&Y, and the result is shown in Figure 1. The frequency of patients with non-motor fluctuations was higher in stages 3 and 4 (64.7 and 66.7%, respectively).

The frequency of NMS fluctuations was compared with the frequency of MS fluctuations. The results are shown in Table 4. No patient presented only NMS fluctuation. Notably, 30 (81.1%) patients had MS fluctuations and, among them, 20 (66.7%) patients also had NMS fluctuations.

**Table 4:** Frequency of motor symptoms and non-motor symptoms fluctuations.

Symptoms fluctuations	WOQ-19 NM<1+	WOQ-19 NM≥1||	Total	p-value
n (%)	17 (45.9%)	20 (54.1%)		
WOQ-19 M<1*	7 (100%)	0	7 (18.9%)	0.002
WOQ-19 M≥1§	10 (33.3%)	20 (66.7%)	30 (81.1%)	

* Absence of MS fluctuations;

+ absence of NMS fluctuations;

§ presence of MS fluctuations;

|| presence of NMS fluctuations.

MS: motor symptoms; NMS: non-motor symptoms.

## DISCUSSION

Fluctuations in NMS are a long-term complication of PD and, in some patients, can be considered as debilitating as fluctuations in MS^13^. In this study, 54.1% of patients presented fluctuations in NMS. Due to the absence of standardized tools for research on fluctuations, the literature data on the prevalence of fluctuations vary widely according to the methodology used and the population studied.

Very broad frequencies, ranging from 19^14^ to 100%^13^, have already been described in the previous studies. These studies did not use the WOQ-19 to assess the fluctuations of NMS, which could explain the marked difference found. More recent studies, using WOQ-19, showed frequencies ranging from 38.3 to 49.4%^15–17^ in the study populations, closer to those found in this study. WOQ-19 is recommended by the Movement Disorders Society as a useful tool for screening the fluctuations^18^.

The cohort of Picillo et al.^15^ followed 47 patients for 4 years from the diagnosis of PD and pointed out the female sex as the major risk factor for the development of NMS fluctuations, regardless of the age of onset, levodopa dose, and MS and NMS at the time of diagnosis. This study showed a concordant result: the frequency of fluctuation of the NMS among women was 90.9%, which was more than double the frequency found in men (38.5%).

Among the NMS, anxiety (35.1%) was found to be associated with the highest frequency of fluctuation. It was also the most common non-motor fluctuation in women and men, along with mood changes in men. Van Der Velden et al., in a systematic review, calculated the weighted average of the frequency of anxiety from eight studies and reported it as being 35.4%. The neuropsychiatric category has been identified as the most common NMS fluctuations^19,20^, with anxiety being the most frequent one, in general^13,15^ and in both sex^15^, with a greater impact on quality of life^20^. The proposed explanation for mood-related fluctuations lies in the same pathophysiological basis of motor fluctuations: the degeneration of dopaminergic neurons was involved in the regulation of mood through the mesocortical and mesolimbic pathways^20^.

Among the patients diagnosed with NMS fluctuations, 14 (70%) of them were in the group whose diagnosis of PD had been given before the age of 54 years. Several studies indicate that the earliest age of onset of PD is associated with more frequent development of fluctuations in symptoms^4,21–23^. One possible explanation is that younger people have greater brain plasticity, which may facilitate maladaptive neuroplasticity responses caused by both progressive degeneration of dopaminergic neurons and non-physiological dopamine pulsatility^24^. The degeneration of the neurons is progressive and, therefore, worsens over the years, being associated with a higher prevalence of the therapeutic complications^25^. Therefore, a higher frequency of fluctuation in patients with longer disease duration has also been found in studies^4,14,21^. In this study, the result was not different, since the majority (70%) of patients with NMS fluctuations were in the group with the longest disease duration (>6 years).

The frequency of patients with non-motor fluctuations was higher in the moderate and severe stages of the disease (64.7 and 66.7%, respectively). Studies have shown significantly higher scores on H&Y stages in patients with non-motor fluctuations^21^, as well as patients with mixed fluctuations with more severe MS, accessed by the H&Y scale^16^. The discomfort caused by NMS fluctuations was also correlated with disease severity^13^.

The presence of fluctuations in NMS is strongly associated with fluctuations in MS. In the L. Brun et al.'s^14^ cohort with 303 patients, only 14% of patients presented fluctuations only in NMS, and in the study by M. Seki et al., this number was even lower (7%)^16^. The result of this research showed that, in the study sample, no patient (0%) presented only fluctuation of NMS. Among those with MS fluctuations, 66.7% also had fluctuation of NMS, a very close result compared to that reported in the study by Rodríguez-Violante M et al., where the calculated frequency from the published data revealed that 66.1% of patients presented fluctuations of NMS among those who had motor fluctuations^17^. In the latter study mentioned, patients with NMS fluctuations had the worst quality of life scores compared to those with mixed or MS fluctuations only, suggesting that NMS fluctuations have a greater impact on patient's quality of life than MS fluctuations.

This study has some limitations. Although recommended by the Movement Disorders Society, the use of WOQ-19 was one of the limitations, because it is still a very superficial tool for evaluating fluctuations, assessing just the presence of them, without measuring frequency and severity of symptoms. With the increasing recognition of the importance and impact of NMS fluctuations, new and more appropriate tools are expected to be tested and validated, and finally be available for use in clinical practice. In addition to this limitation, a small sample size, selected for convenience, in a hospital-based study, which is not representative of all PD populations, does not allow the results to be extrapolated and validated externally beyond the study population.

This study showed that, in the study population, approximately half of the patients presented NMS fluctuations, being this frequency much greater in the female sex. A higher frequency was present in patients with earlier age of diagnosis, longer duration, and greater severity of disease. The symptoms that fluctuated most were those of the neuropsychiatric category, especially anxiety. It is extremely important to recognize fluctuations of this category, since, for example, the recognition of anxiety as fluctuation will result in treatment with dopaminergic replacement, and not with antidepressants. Finally, there were no patients with fluctuations only in the NMS.

These findings point to the importance of recognizing fluctuations in patients with PD in the study population, since these may not be spontaneously mentioned by the patient, who is remaining unnoticed, undiagnosed, and not treated by the neurologist, representing a not insignificant aggravating factor in the patient's quality of life.
